# Feeding Young Children in Food Insecure Environments: A Photovoice Study in Kenya's Drylands

**DOI:** 10.1111/mcn.70247

**Published:** 2026-10-15

**Authors:** Monica Nthamba Ng'ang'a, Esther Anono, Hazel Odhiambo, Estelle M. Sidze, Faith Thuita, Calistus Wilunda, Dickson Amugsi, Bonventure Mwangi, Ruth Muendo, Florence Sipalla, John Ebei, Chessa K. Lutter, Albert Webale, Teresia Macharia, Elizabeth Kimani‐Murage, Valerie L. Flax

**Affiliations:** ^1^ RTI International Nairobi Kenya; ^2^ African Population and Health Research Center Nairobi Kenya; ^3^ Department of Public & Global Health University of Nairobi Nairobi Kenya; ^4^ Mercy Corps Nairobi Kenya; ^5^ RTI International Durham North Carolina USA; ^6^ International Rescue Committee Nairobi Kenya

**Keywords:** Africa, food access, food availability, food security, Kenya, photovoice, wasting

## Abstract

Child wasting remains a global public health challenge, with persistently high levels in dryland regions such as Turkana and Samburu Counties in Kenya. Food scarcity is linked to spikes in wasting prevalence in these counties, but little is known about women's lived experiences of food insecurity and how they perceive its relationship to child wasting. A total of 126 women across diverse livelihood zones in Turkana and Samburu Counties participated in a photovoice activity as part of the Nawiri mixed‐methods longitudinal study. The women took photos based on four prompts related to food security and discussed their photos in groups of six. Transcripts of the discussions were analyzed thematically. In both counties, most households relied on local markets, shops, and kiosks for food. Diets were dominated by maize year‐round but included wheat products, meat, a limited number of vegetables, and milk in times of plenty. During dry periods, households coped with food insecurity by adding liquids to stretch food, reducing the number of meals per day, and consuming wild fruits. This partially addressed hunger, but participants said that lack of food and poor‐quality food contributed to child wasting. Barriers to food security included low and unreliable incomes, issues related to shops (i.e., limited types of food, frequent stockouts, short opening hours, and distance from communities), high food prices, and lack of transport. Participants highlighted the need to increase the availability of fruits and vegetables in shops. The Nawiri program used these findings to adapt multisectoral activities to address these challenges and decrease wasting.

## Key Messages

- Households in Turkana and Samburu Counties relied heavily on markets, shops, and kiosks for food.
- Diets were monotonous, with maize dominating year‐round, but occasionally included wheat products, meat, vegetables, and milk during rainy seasons.
- During droughts, households added liquids to increase food volume, reduced the number of meals, and consumed wild fruits to cope with food insecurity.
- Participants said that lack of food and poor‐quality food during dry seasons led to child wasting.
- Barriers to food security included low income, high food prices, stockouts of food, lack of fruits and vegetables in shops, long distances to markets, and lack of transport.

## 1 Introduction

Child wasting remains a pressing global public health challenge, with persistently high levels reported in dryland regions, such as Kenya's Turkana and Samburu Counties (Young et al. 2021). In 2024, wasting prevalence was 21.5% in Turkana and 17.8% in Samburu (Kenya Ministry of Health 2024), both above the emergency threshold of 15% (IPC Global Partners 2021). Wasting can persist or recur in dryland settings even in the absence of clear emergencies and despite ongoing interventions (Young and Marshak 2018). The conceptual framework for acute malnutrition in Africa's drylands highlights that wasting is driven by multiple factors, with inadequate dietary intake as an immediate cause and insufficient household food security as an underlying contributor (Young et al. 2021).

This study was conducted as part of the first wave of data collection in the Nawiri mixed‐methods longitudinal study in Turkana and Samburu Counties, which was designed to examine the drivers of wasting (Wilunda et al. 2026). This qualitative component of the study complemented survey findings (Amugsi et al. 2026) by examining lived experiences of food insecurity and capturing dimensions of food insecurity that standard measures may miss because of the high prevalence of severe food insecurity in these counties.

Food insecurity is present when a group or population does not consistently have physical, social, and economic access to adequate, safe, nutritious, and culturally acceptable food to meet their dietary needs (Food and Agriculture Organization and World Food Program 2010). Survey data from the Nawiri longitudinal study showed that food insecurity was not directly associated with child wasting in either county (Amugsi et al. 2026). A review by the Food and Agriculture Organization found that food insecurity was associated with wasting in only 3 out of 15 studies in low‐ and middle‐income country contexts (Maitra 2018), although it is clear that wasting prevalence spikes during periods of food scarcity. In the Nawiri longitudinal study, the lack of association is likely because there was little variation in the food security indicator. All households experienced some form of food insecurity and ≥ 85% of households in Turkana and ≥ 73% of households in Samburu experienced severe food insecurity across survey waves (Wilunda et al. 2026). Analysis of factors contributing to food insecurity in these counties showed that caregivers having any education and household poultry ownership were associated with reduced odds of food insecurity, while lower levels of wealth, more shocks, and drought periods were associated with increased odds of food insecurity. Pastoral households in Turkana and agropastoral households in Samburu had greater odds of food insecurity. Several of these findings align with a review of factors related to food insecurity among dryland pastoralists and agropastoralists (Stavi et al. 2022), but do not provide essential contextual information and lived experiences of food insecurity in these contexts or how community members perceive the linkages between food insecurity and child wasting.

Photovoice actively involves community members in research by allowing them to capture images that reflect their daily realities, experiences, and needs (Wang and Burris 1997). This method empowers participants to analyze and interpret their own photographs, offering personal narratives that highlight critical issues, especially those affecting marginalized groups (Liebenberg 2018; Mitchell and Sommer 2016). Previous studies using photovoice to explore food insecurity experiences in sub‐Saharan Africa have focused on adolescents in Ghana and urban poor in Kenya (Osogo et al. 2025; Posey et al. 2024), but not in dryland settings, such as northern Kenya. This paper explores how community members navigate food insecurity in Turkana and Samburu Counties, a context where households face numerous shocks and stresses, by investigating where participants obtain food, how food availability varies across seasons, factors that make it difficult to achieve food security, and how food availability and food insecurity relate to child wasting.

## 2 Methods

### 2.1 Study Design

The Nawiri longitudinal study was a mixed‐methods study that included three qualitative methods (photovoice, participatory community causal mapping of factors influencing acute malnutrition, and qualitative focus group discussions, in‐depth interviews, and key informant interviews) to provide community input and explanations for findings from the quantitative component. This analysis reports on the photovoice findings. By capturing images representing their realities and participating in the photovoice process, participants in our study were able to have meaningful group discussions centered on food insecurity in their communities and how it relates to wasting. These visual narratives served as a foundation for deeper conversations on challenges within the context of Turkana and Samburu Counties.

### 2.2 Study Site

Turkana and Samburu Counties are located in Kenya's arid and semi‐arid lands (ASALs), which are prone to droughts, floods, and livestock disease outbreaks (Lutta et al. 2023). In 2022, 83% and 72% of the population in Turkana and Samburu, respectively, lived below the poverty line (Kenya National Bureau of Statistics 2024). Livestock raising is important across livelihoods in Kenya's ASALs and accounts for > 95% of household income. Livestock contribute to food security as a food source (i.e., meat, milk, and blood) and through income from the sale of livestock and livestock products, which can be used to purchase other foods (Scoones 2003).

For the photovoice activity, in consultation with county officials, seven villages from among those included in the longitudinal survey were purposively selected to represent each livelihood type [pastoral, agropastoral, urban/peri‐urban, and fisherfolk (Turkana only)] in each county. Three photovoice sessions were held per village, resulting in a total of 21 photovoice sessions in the study.

### 2.3 Participants

Participants eligible for the photovoice activity were adolescent mothers (10–17 years), young mothers (18–24 years), and adult mothers (25 years and above) with a child under 5 years and willing to provide informed consent. These groups were selected to ensure that mothers of different ages were represented in the sample. Participant mobilization in the selected villages was supported by local leaders such as chiefs, assistant chiefs, and community health promoters. In total, 126 mothers of children < 5 years (72 Turkana, 54 Samburu) participated in the full photovoice cycle. Within each selected village, three separate photovoice sessions were held, one per age group. Each photovoice session had six members who were divided into two groups of three participants to take photos, but who came together as a whole group for the training and discussion.

### 2.4 Data Collection

Photovoice data were collected from May 10 to July 31, 2021, which was during the dry season. Research assistants from the counties who spoke the Turkana, Samburu, and Swahili languages participated in a 1‐week training to learn how to train participants and lead the steps of the photovoice process. Training for photovoice participants lasted half a day. This included a discussion of the photovoice method, research topic, and the specific prompts for the photos; practicing how to operate a camera and take photos; learning about photo ethics and safety precautions related to taking photos; ensuring that no faces were included in photos; asking people for permission before taking photos; taking photos as they were going about their normal routine; selecting photos for printing; meeting as a group to develop posters and captions for the printed photos; and discussing the photos as a group. Community dissemination of photovoice photos and captions was included in the dissemination activities for the first wave of the Nawiri longitudinal study. The prompts for participants' photos related to food security were:

- A place where you get or buy food;
- The types of food your family eats during seasons of plenty or when money is available;
- The types of food your family eats during lean seasons or when food prices are high or money is unavailable; and
- Things that make it difficult or easy to attain your food basket.

We purchased five digital cameras (Bright Tech Sony DSC W830) per county. Each camera was shared by three participants in the same age group who lived near each other. Working in a small group allowed participants to remind one another of the prompts and ensured safety. They were asked to spend 1–2 days capturing photographs that reflected their personal experiences and views on food security based on the prompts. After piloting, we determined this was enough time for participants to take photos. Each participant took a minimum of one photo per prompt. On the day of the discussion, the groups that shared a camera met with a research assistant, who saved their photos to a laptop. The research assistant displayed the relevant photos for each prompt on the laptop one at a time, and the person who took each photo explained what it meant to them. The group of three then discussed which of their photos they felt most represented their experiences in relation to the prompts and selected one for each prompt. The research assistant printed the selected photos and asked the person who took the photo to provide a caption, which the research assistant wrote and attached to the printed photo. The photos from both groups of three were posted on the walls for the photovoice discussion. Photovoice sessions were held in schools or churches in the study communities. The six participants gathered with the research team as a group to reflect on the photos, discuss the meaning of each photo, and share their interpretations. The discussion questions followed the SHOWED method using a discussion guide (Supplemental Materials) (Wang 1999). Each group also collaboratively identified and discussed key themes or topics that repeated across photos. The discussions about the photos were audio‐recorded and facilitated by research assistants. A research assistant took notes to help link quotes to specific photos during the analysis. Each discussion lasted 2–3 h.

### 2.5 Data Analysis

Audio recordings and photographs were labeled with the participants' livelihood zone and county. After saving photos to a secure laptop and backing them up, they were deleted from the camera. Transcription was conducted by skilled transcribers from the local community, selected for their prior experience with verbatim transcription, fluency in the local language, and ability to accurately translate content into English. Accuracy of transcripts was checked against the recordings by four individuals fluent in the local languages. The research team and a professional qualitative coder independently applied codes based on the subthemes related to each prompt that emerged during the discussions of the photos. The analytic approach was primarily inductive, but was also informed by the study prompts. The textual analysis was linked to specific photos and used to identify quotes from participants that illustrated key themes or subthemes. The transcripts were imported into QSR Nvivo 12 software for coding, and the analysis was carried out using a thematic approach (Clarke and Braun 2017).

### 2.6 Ethics and Reflexivity

Ethical approval for the study was obtained from Amref Ethical and Scientific Review Committee (P905‐2020), and a research permit was obtained from the National Commission for Science, Technology, and Innovation of Kenya. Additionally, a reliance agreement was signed between the institutional review boards at the African Population Health Research Center and RTI International. Written informed consent, including signed photo release, was obtained from participants, granting permission for the use of their photos in study‐related reports and publications.

The research team tried to minimize power differentials that may have influenced the photovoice process and discussions by grouping women of the same age for discussions and to share cameras, using local research assistants to lead the training and discussions, and including only women as participants in the photovoice study. These strategies helped to ensure that participants felt comfortable talking about their photos and speaking up during the discussions. In addition, each participant had an opportunity to discuss at least one of their photos in depth before further group reflection and discussion.

For most of the participants, this was the first time they used a camera, and they felt that learning how to take a photo was an empowering skill. Once their photos were printed, an emotional shift occurred because they could see their own lived realities, which sparked personal reflection. They also saw how the photovoice methodology and sharing their stories could be a tool for advocating for help from leaders to address systemic issues that they could not fix on their own.

The analysis was led by Kenyan members of the research team with deep knowledge of these two counties. The research team relied on the themes and sub‐themes from the discussions to guide the analysis and reflected on the process of selecting photos for inclusion in the paper in relation to themes.

## 3 Results

The key themes were related to where households get or buy food, types of food available and seasonal variation in availability, and barriers to food security. Summaries of the sub‐themes within each theme are presented, highlighting variations by county, season, and livelihood, where relevant.

### 3.1 Where Households Get or Buy Food

Through the photos and discussions, participants described three sub‐themes related to where they get or buy food: markets, shops, and kiosks; livelihood‐related food production; and food aid (Table 1).

**Table 1:** Where households get or buy food.

Findings	Quotations	Images
Markets, shops, and kiosks		
Purchase of mainly dry food in kiosks or shops	*This is where we buy food for our households. We get everything here. It is in our village*. Fisherfolk mother, Turkana	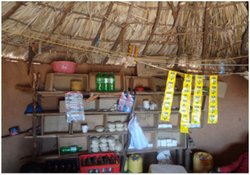
Local shops and kiosks served as the primary source of staples, like maize and beans	*This is the shop where we buy food for the household. The common and available foods in the shop are usually…maize and beans*. Agropastoral mother, Samburu	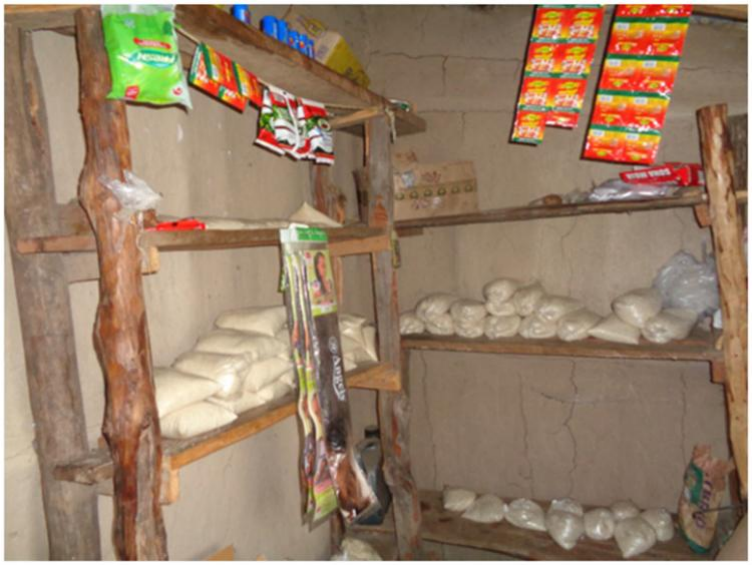
Wider variety of food in peri‐urban shops, although not always available	*This shop provides nutritious foods that we need for our children, such as milk, meat, potatoes, bananas, avocados, and pawpaws. These foods supply essential nutrients that support healthy growth and development, strengthen the blood, and promote overall good health. However, not all the desired food varieties are always available*. Peri‐urban mother, Samburu	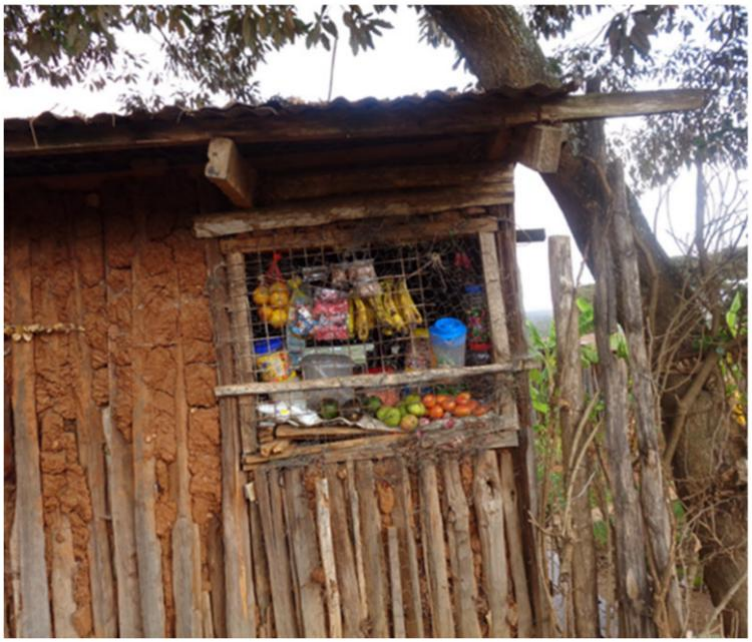
Food production		
Pastoral and agropastoral communities had access to meat	*This is goat meat. When it gets finished, it can lead to malnutrition*. Agropastoral mother, Turkana	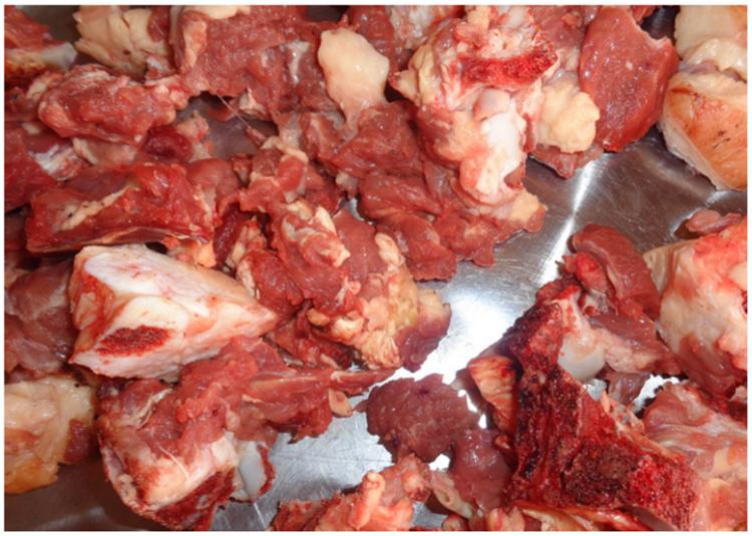
Pastoral and agropastoral families had access to milk during the rainy season	*During the rainy season, there is milk from livestock that you can give to your child to improve their health and sell the surplus. When you sell a cup of milk, you can buy at least some food like cooking fat, maize flour, rice, beans, and salt*. Pastoral mother, Samburu	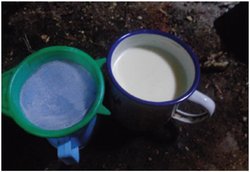
Agropastoral and peri‐urban families ate their own vegetables during the rainy season	*This is a farm where we cultivate food such as maize, beans, green peas, sukuma wiki (collard greens), and spinach during the rainy season*. Agropastoral mother, Samburu	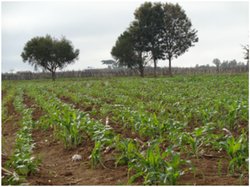
Fisherfolk families in Turkana ate fish they caught, although it was not a preferred food	*This is called nangaria. It is a light food and roasted fish. We eat this food when there is no money*. Fisherfolk mother, Turkana	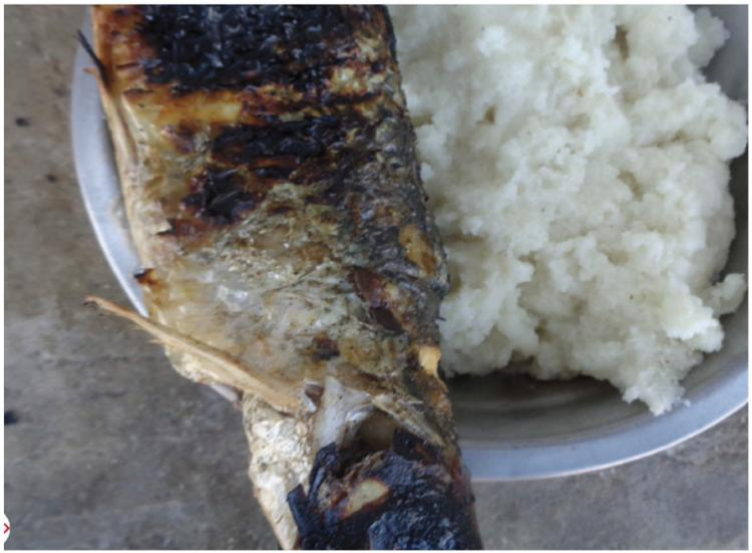
Food aid		
Reliance on poor quality food aid during drought	*We get food aid from the government to support during drought, which is white maize. You lack the money to mill this maize to flour. Also, the maize is dirty and infested with black flies (weevils), which have destroyed most of it. You just go wash the maize and immerse in the pot and the children eat with flies in it*. Agropastoral mother, Samburu	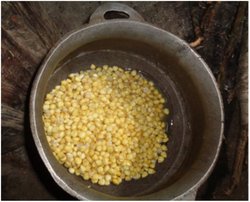

#### 3.1.1 Markets, Shops, and Kiosks

In both counties, most households relied on markets, shops, and kiosks for food purchases, regardless of their livelihood or the season. Markets were linked to the livestock trade, occurred weekly, and offered an opportunity to buy food in bulk and find a slightly wider variety of foods, but they were located far from most homesteads. Shops and kiosks were the most frequent source of food. They provided mainly dry goods in smaller quantities and also served as a source of food on credit. However, frequent stockouts at shops affected food availability. For many households in Turkana, the distance to any market, shop, or kiosk was far and transport was unreliable, leading women to adopt an irregular pattern of food purchase. In both counties, dry foods, such as oil, sugar, maize, beans, and rice, were the most commonly available items across markets, shops, and kiosks. In Turkana, participants took photos of and mentioned the presence in shops of high‐energy snacks, such as biscuits, *mandazi* (doughnuts), and sweets. In both counties, fresh produce was mostly found in peri‐urban areas, but its availability was limited, even in those areas, due to its perishability. The few available fresh food items included potatoes, bananas, cabbage, avocados, and onions, and sometimes tomatoes and *sukuma wiki* (collard greens). In both counties, participants highlighted the need to improve the variety and quantity of nutritious food items, such as vegetables, fruits, and soft foods for children, in local shops to enhance availability.

#### 3.1.2 Food Production

In both counties, livelihood‐related food production was another major source of food. In Turkana, agropastoral households supplemented food from markets and shops with what they produced themselves. The fisherfolk community included fish as part of their diet, while pastoral communities had access to milk and animal blood. Across livelihoods, food production, availability, and variety were greatest during rainy periods or during the early part of the dry period.

In Samburu, agropastoral and peri‐urban communities obtained food from their own farms during the rainy season, supplemented by purchases from shops, markets, and groceries. Some agropastoral community members also relied on nearby rivers for fish, including *omena* (silver cyprinid). During the rainy season, animal source foods were available to agropastoral and pastoral households from their own livestock.

#### 3.1.3 Food Aid

During drought periods, many families, especially in pastoral and agropastoral livelihoods in both counties, also depended on food aid as a critical food source. Participants explained that food aid was essential to keep children alive when families had no income and no other way to obtain food. Participants said that the quality of food aid they received was low and mentioned that the maize frequently contained insects.

### 3.2 Types of Food Available and Seasonal Variation in Availability

Based on the discussions, sub‐themes related to types of food available and seasonal variation in availability were: foods participants' households eat during times of plenty and lean seasons, strategies they use to cope when they have limited or no food, and how food insecurity relates to child wasting (Table 2).

**Table 2:** Types of food available and seasonal variation in availability.

Findings	Quotes	Images
Foods eaten during times of plenty		
Families preferred meals that included wheat products, meat, *githeri*, tomatoes, onions, and potatoes when they had money	*This is the food [wheat flour used to make chapati or pancakes] we eat when there is money to purchase*. Pastoral mother, Turkana	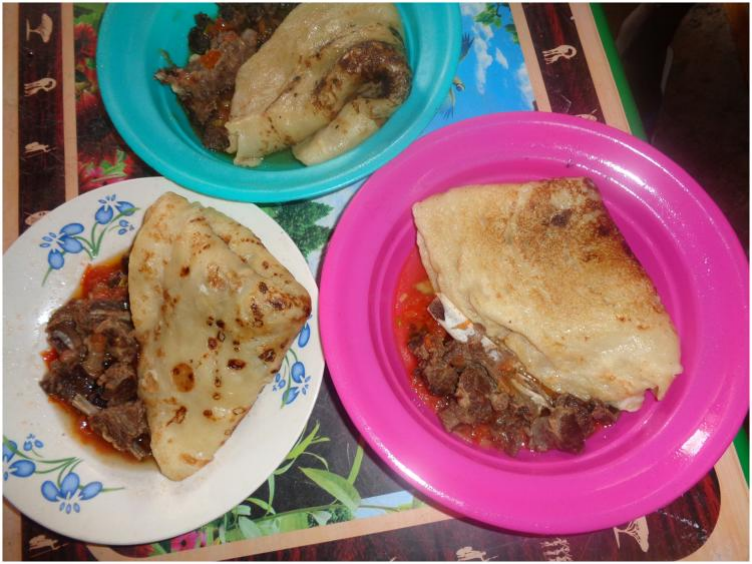
	*This is meat and potato stew. It is the kind of food we eat when the season is good. It is healthy and confers beneficial value to the body*. Pastoral mother, Samburu	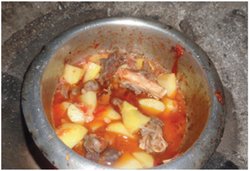
Foods eaten during lean seasons or drought periods		
Preparing foods considered to have low nutritional value	*I can cook porridge consisting of maize or sorghum, but it is not nutritious, because it has only salt. There is no inclusion of something like Blue Band [brand of margarine] or sugar to give you energy when you drink this porridge*. Peri‐urban mother, Turkana	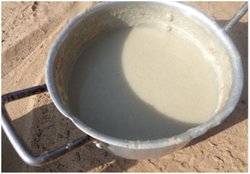
Consuming food of plain white maize to prevent hunger	*When you do not have money, especially when there is drought, there is nothing. Even getting food from the market is a challenge, as it is not available. Even the food vehicles are no longer coming to the market to bring food. What can we do? There is only white maize, which we consume when it's available rather than stay hungry. It is better to cook that maize and give it to your children. They cannot withstand hunger*. Pastoral mother, Samburu	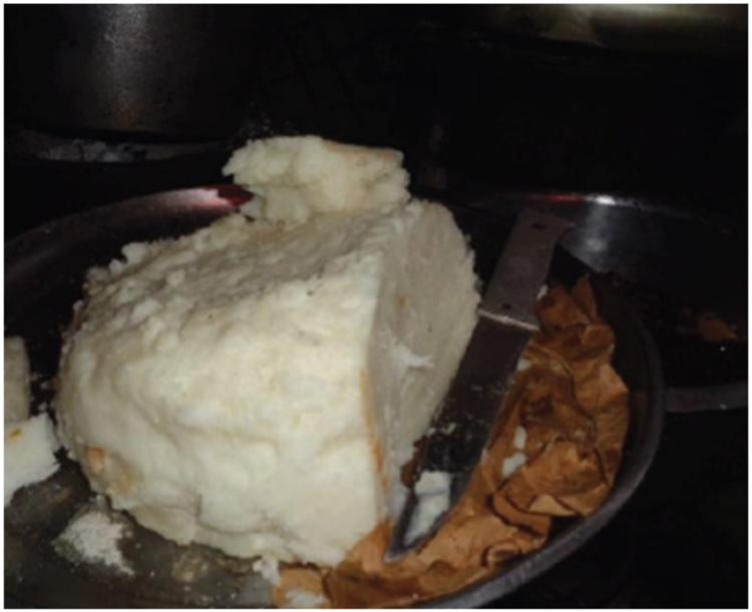
Coping strategies during drought periods		
Using liquids to increase volume of meals	*This is wheat flour, maize, and alcohol. When we want to eat that food, we will mix that alcohol with food due to lack of oil. I will divide the food and cook it with the alcohol and we consume it, including children*. Pastoral mother, Turkana	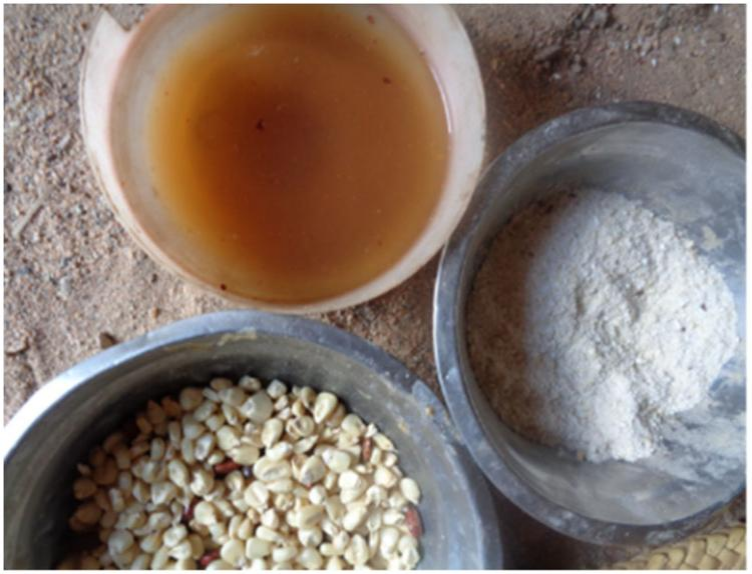
Reduced quantity and frequency of meals	*All family members eat only this food when rice is enough, but if the quantity is small, you can give it only to the children and others stay like that [without food]*. Agropastoral mother, Turkana	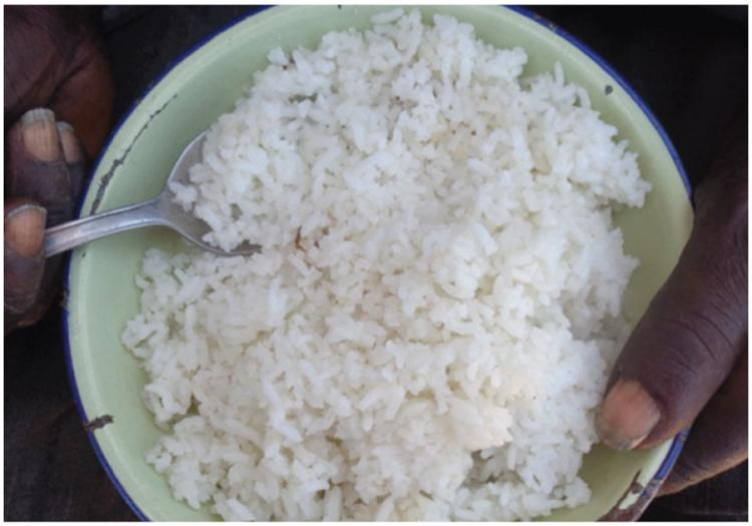
Pastoral and agropastoral families consume wild fruits as a survival food	*We consume this local fruit called elmach when we do not have money to buy food. Lack of something else to eat is what makes us eat this wild fruit…It has driven our children to malnutrition*. Agropastoral mother, Turkana	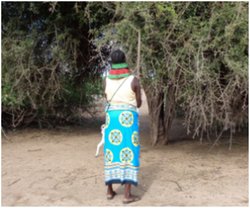
	*This is ‘lordo’, a wild fruit. We mix it with blood and drink when we do not have money*. Pastoral mother, Samburu	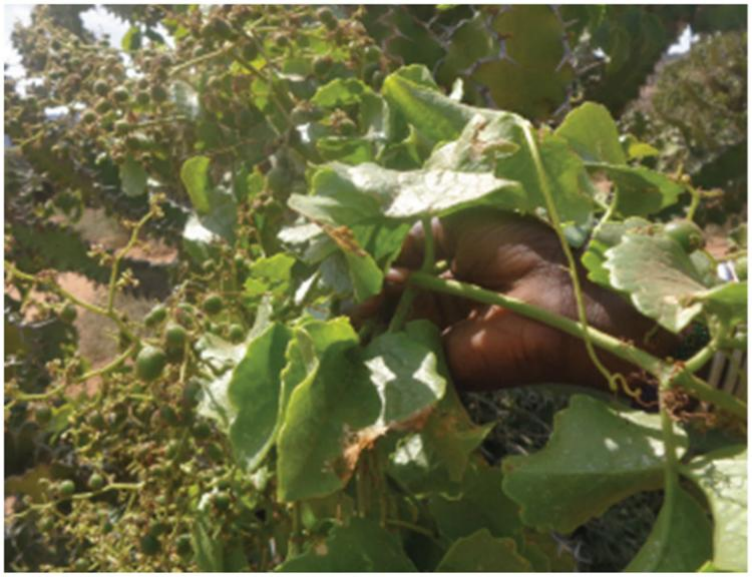
How food insecurity relates to child wasting		
Lack of food and low‐quality food contributes to wasting	*This is food for the young children here. This is porridge, which is watery…. They just drink so that the body becomes active but there is no nutritional value*. Agropastoral mother, Turkana	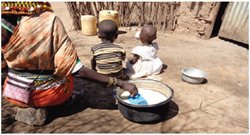
	*During drought, we just prepare this porridge with no cooking fat. It's just water, and not even salt is added. When a young child takes this porridge, they might get kwashiorkor*. Agropastoral mother, Samburu	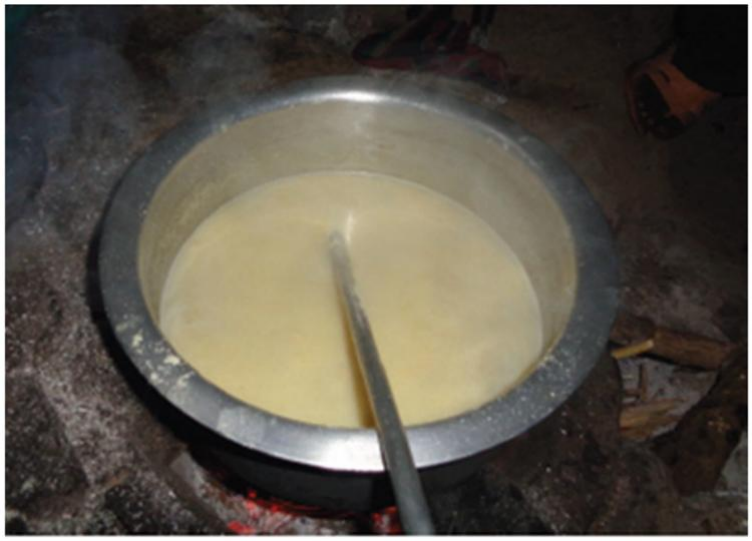
Lack of milk when livestock are away from households during dry season contributes to wasting	*During drought when the livestock have gone to satellite camps, there is no milk*. Agropastoral mother, Samburu	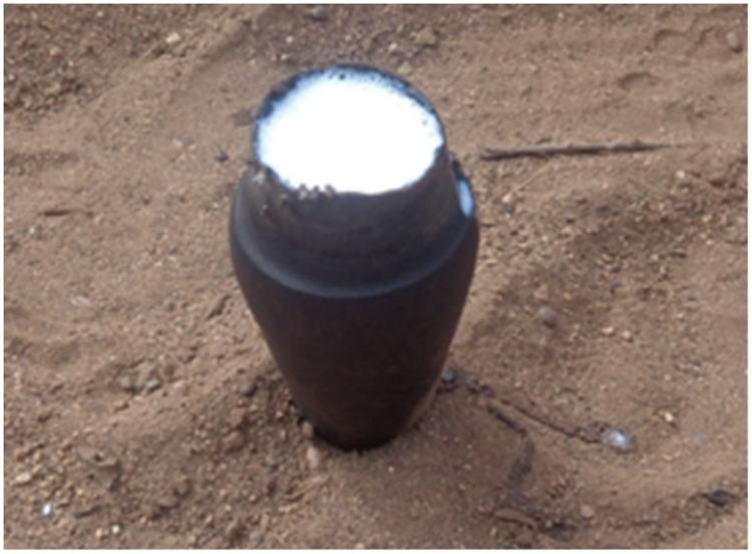

#### 3.2.1 Foods Eaten During Times of Plenty

In both counties, many households across different livelihoods consumed monotonous diets, primarily centered around maize or maize meal, which was the most available and affordable option during both times of plenty and lean seasons. However, during the rainy season or times of plenty, a wider variety of foods were available in markets and on farms. Household members also tended to have more paid employment and greater income from livelihood activities, allowing them, at times, to purchase their preferred foods. In both counties and across livelihoods, when they had money, most families preferred meals that included wheat products, including chapatis, *mandazi* (fried dough), and spaghetti; meat; *githeri* (mix of beans and maize); tomatoes; onions; and potatoes. Participants explained that they preferred these foods because they were nutritious. Pastoral households had access to milk, meat, and blood from their own herds during times of plenty. Agropastoral households included foods like beans, peas, potatoes, *sukuma wiki*, spinach, and tomatoes from their own production in their meals during the rainy season. Peri‐urban households had greater access to fruits and vegetables in shops during the rainy season.

#### 3.2.2 Foods Eaten During Lean Seasons or Drought Periods

In both counties, during lean seasons or drought periods, many households experienced shortages in food quantity and variety, largely due to high food prices and low incomes. Across livelihoods, the most common foods during the dry season were plain white maize meal porridge, plain boiled maize, soft *ugali* (stiff maize porridge), and plain white rice. These foods were not considered to be nutritious but were eaten to prevent hunger.

#### 3.2.3 Coping Strategies During Drought Periods

During dry periods or droughts, families had to stretch limited food supplies. To cope with food scarcity, common strategies included cutting down the number of meals from three to one per day, and in severe cases, not eating for the whole day. Children were prioritized, while adults ate less or went to bed hungry. Food was prepared without any added salt, cooking oil, or margarine. Foods made of maize were stretched by diluting them using water or milk, and in Turkana, sometimes alcohol, to increase volume. Alcohol was used when no cooking oil was available.

In both counties, wild fruits such as *lordo (Solanum quitoense lam)* in Samburu and *edapal (Dobera glabra juss)*, *elmach (Balanites pedicellaris), edome (Cordia sinensis)*, *esekon (Salvadora persica)*, *edong (Spondias dulcis)*, and *apinet* (powder from palm fruit or *Hyphanene coriacea*) in Turkana, were commonly consumed during periods of food scarcity. However, caregivers viewed them as having minimal nutritional value, and the fruit *edapal* required up to 3 days of preparation before it was safe to eat. Additionally, some caregivers reported cases of diarrhea in young children following the consumption of wild fruits.

#### 3.2.4 How Food Insecurity Relates to Child Wasting

Participants across livelihoods explained that feeding children watery porridge, plain maize porridge, boiled maize, or plain white rice was to prevent hunger but did not have much nutritional value and eating these foods alone would lead children to become wasted. Pastoral and agropastoral mothers said that malnutrition rates among children are elevated when it is dry because livestock are taken to pasture far from households and children do not get milk and because livestock die or are unsellable so there is no money for food.

### 3.3 Barriers to Food Security

For the prompt related to things that make it easy or difficult to attain their food basket, participants exclusively took photos of barriers to food security or things that make it difficult to attain food. Sub‐themes that emerged included inadequate household income and high food prices; distance to markets, poor transport infrastructure, and insecurity; and issues related to shops, such as high food prices, short operating hours, and food stockouts (Table 3).

**Table 3:** Barriers to food security.

Findings	Quotes	Images
Inadequate income and high food prices		
Low income from women's casual labor	*This is just causal work. It is work that you carry firewood and walk all over and at times you can get a person who buys it. When there is no one to buy, you take it back home*. Agropastoral mother, Turkana	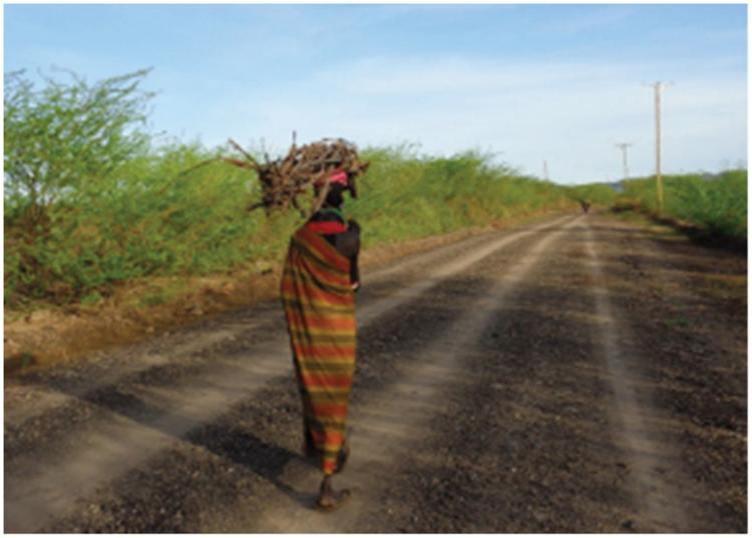
Lack of money to meet basic food needs	*Money makes it easy to attain your food basket, because when you have money you can buy a variety of foods, and when you do not have money, you cannot buy a variety of food*. Pastoral, mother, Samburu	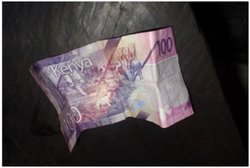
Rising food prices during the dry season significantly impacted ability to buy food	*This is where we buy our food, although the prices in this shop are very high*. Pastoral mother, Turkana	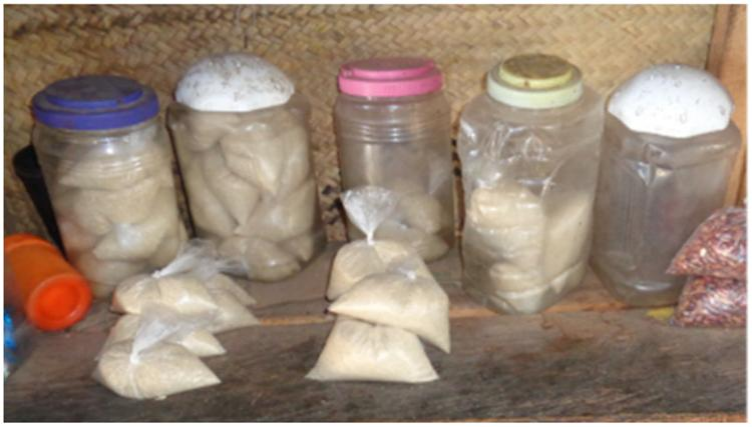
Distant markets, poor infrastructure, and insecurity		
Distance limited access to shops and markets	*I usually go to Lodwar but it may be far for me. When I decide to go there, I will have to spend many days on the way and when I come back, I find that the children are in a bad state. I find that the young children have eaten palm fruit, and they have diarrhea*. Pastoral mother, Turkana	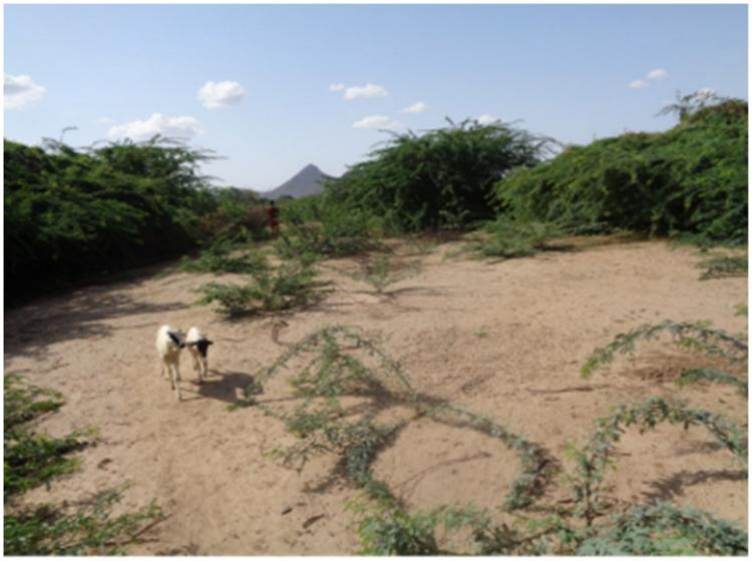
Poor roads and lack of public transport limited food access	*The means of transport is poor due to the bad state of the roads. If the government can try to construct roads for us then the prices of food will be cheaper, so the government can help*. Peri‐urban mother, Samburu	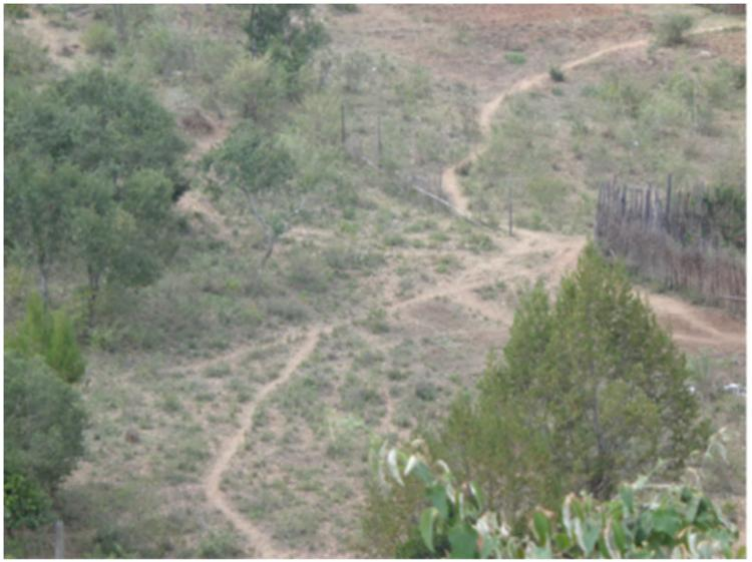
Barriers related to shops and kiosks		
Shops frequently ran out of stock	*Usually, I purchase food here but the food is out of stock now. I consume a wild fruit called edapal as I wait for the food to be stocked back in this shop*. Agropastoral mother, Turkana	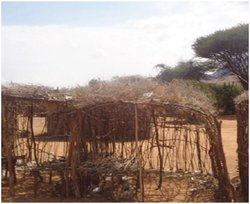
Shops closed early	*If you engage in casual jobs whereby you come [home] late, you find that the shop is closed, and there is nowhere else to go and buy food*. Peri‐urban mother, Samburu	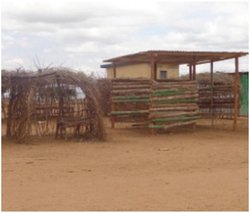
No fruits or vegetables in shops	*There are no fruits or food that I want in this shop. This is affecting what we provide for our children*. Agropastoral mother, Turkana	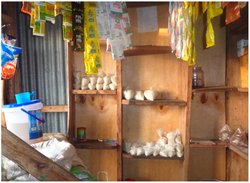

#### 3.3.1 Inadequate Income and High Food Prices

A major obstacle to food security was that most community members relied on livelihood activities that generated insufficient income to meet their basic needs. In many households, women engaged in casual work (e.g., collecting and selling wood, agricultural labor) or petty trade, which was the only source of cash income during dry seasons, but was often low‐paying or unreliable. In both counties, low household incomes coupled with rising food prices during the dry season significantly impacted the amount and types of food they could buy. As purchasing power declined, many families were forced to buy only what was affordable, without regard for the nutritional needs of their household members. Participants explained that many families could not afford basic ingredients like cooking oil, fat, salt, or sugar, which they consider to be essential for enhancing the energy content of meals, especially for children.

#### 3.3.2 Distant Markets, Poor Infrastructure, and Insecurity

As previously mentioned, in both counties, markets and shops were often situated far from households, and reliable transportation was scarce or non‐existent. Many people, especially mothers, had to walk long distances to access markets or shops. As a result, mothers commonly adopted a routine of making weekly or even less frequent food purchases for their families. Poor road network connectivity significantly limited access to a variety of foods, particularly in pastoral communities. In Turkana, in some communities, shops could only be reached by crossing a river, which became a barrier to food access during the rainy season. Additionally, pastoral households frequently struggled to obtain food on credit, further limiting food access. In Samburu, among pastoral communities, insecurity, characterized by raids or fighting between different communities, also restricted access to food.

#### 3.3.3 Barriers Related to Shops and Kiosks

Participants described a few barriers related to purchasing food at shops and kiosks. While shops remained an important source of food, they frequently ran out of stock because shop owners only purchased new supplies of food when they had enough money to do so. Women who worked doing casual labor complained that shops were often closed by the time they came home from work, so they could not buy food. Participants also complained that shops did not sell fruits or vegetables, which limited the types of food they could provide for their children.

## 4 Discussion

This photovoice study shows how participants' lived experiences of seasonal variations in food availability and access, market dependence, and issues specific to certain livelihoods shape their perceptions of the relationship between food insecurity and child wasting in Turkana and Samburu Counties. In both counties, markets, kiosks, and shops were the main sources of food, with maize meal dominating diets year‐round. Diets were inadequate and monotonous during dry seasons but included wheat products, meat, a limited number of vegetables, and milk during rainy seasons. During dry periods, households coped with food insecurity by adding liquids to stretch food, reducing the number of meals per day, and consuming wild fruits. Previous research in Turkana has shown that people associate consumption of wild fruits with food scarcity and many have unfavorable opinions about eating them, which aligns with the findings of this photovoice study (Oduor et al. 2023). Eating watery, maize‐based meals and wild fruits was a way to partially address hunger, but participants said that lack of food and poor‐quality food contributed to child wasting. Households faced many barriers to food security. Most households had a single, unreliable source of income and did not have enough money for food when prices increased during the dry season, limiting economic access to food. Long distances to markets, lack of transport, conflict (especially in Samburu), stockouts of food in shops, and early closing hours also restricted food access and food security.

These photovoice findings provide context for the results of the Nawiri longitudinal study quantitative analysis by showing how lower levels of wealth, shocks, droughts, and pastoral and agropastoral livelihoods are related to severe food insecurity and child wasting (Wilunda et al. 2026). Previous research has shown that household food insecurity scales, like the Food Insecurity Experience Scale used in the Nawiri longitudinal study, are useful for making population‐level estimates of household food insecurity over time, but do not capture the details of household food insecurity experience (Coates et al. 2006). This suggests that both quantitative and qualitative measures of food insecurity are needed to understand the extent of the problem along with the context‐specific experiences of food insecurity that can be used for designing programs (Johnson et al. 2021). Like other studies of food insecurity globally, our qualitative photovoice data shows that households experiencing food insecurity mention inadequate food quality, insufficient food quantity, hunger, and links between food insecurity and weight loss or, in the case of our study, wasting (Coates et al. 2006).

Several aspects of the results from this photovoice study have been highlighted in other studies in African drylands. Many households in the study counties were located far from shops and markets, and without reliable transport, mothers often walked long distances and adopted weekly purchasing routines to obtain food for their families. According to a study conducted in Kenya's drylands, distant markets are critical for food supply in these regions, especially during droughts when local production falls (Huho and Mugalavai 2010). However, poor infrastructure and high transportation costs limit access to these markets. High prices mentioned by participants in this study align with a recent analysis in seven sub‐Saharan African countries that include drylands, which found that food is more expensive in dryland areas and a nutrient‐adequate diet is not affordable for most dryland households (Hobbs et al. 2023). In terms of nutrient adequacy, participants' photos in this study showed that a minimal number of dry food items were sold in shops. Fresh fruits and vegetables were more accessible and available in peri‐urban areas, but their availability was mostly limited due to challenges of perishability. Participants emphasized the need to increase both the variety and quantity of nutritious foods in local shops to improve the quality or nutrient adequacy of the food they give their children.

Some of our findings align with those of other photovoice studies related to food insecurity in sub‐Saharan Africa and other remote settings, although the contexts of these studies vary considerably. Unstable jobs or lack of employment and lack of money were mentioned as barriers to food security in photovoice studies in Ghana and among remote communities in Australia (Chappell et al. 2024; Posey et al. 2024). Distance to places to buy food, transport issues, and infrequent trips to purchase food were also described in the study in Australia (Chappell et al. 2024). High cost of food, which limited the ability to buy preferred foods, was discussed in remote communities in northern Canada (Lardeau et al. 2011). The other photovoice study in Kenya, conducted in an urban slum area, also found that children were often fed only a cup of porridge a day, although there was a wide variety of foods available in the slum area compared with Turkana and Samburu (Osogo et al. 2025). These commonalities across varied remote and other African settings show that some aspects of food security experiences are the same despite differences in context. However, the severe lack of food and link to wasting shown in our study were not present in the other studies.

Previous research has shown that the peak periods of acute malnutrition among children across the African drylands occur during the hot months and during the months with the greatest rainfall (Venkat et al. 2023). The hot, dry months are when food for humans and fodder for animals is not available, and the rainy months are when agricultural products have not yet been harvested. Our photovoice data were collected during the dry months and indicated that households lacked food during this time of year, and mothers were concerned about feeding their children a diet of watery maize porridge or plain maize. They considered these foods to have no nutritional value or to be indigestible (e.g., plain maize) for young children but provided them to their children to prevent hunger. They also talked about how eating only these foods could cause acute malnutrition in children. These findings also align with the community causal mapping exercise we conducted as part of the Nawiri longitudinal study, which showed that community members considered lack of food or lack of nutritious food to be a strong contributor to acute malnutrition in children in both counties (Odhiambo et al. under review).

### 4.1 Study Strengths and Limitations

Key strengths of our study are the use of a participatory method, inclusion of women of different ages, holding separate photovoice discussions by age group to address power differentials, and inclusion of different livelihood groups. Our study had some limitations. We conducted the photovoice sessions during the dry season, which made it easy for participants to take relevant photos when food insecurity and wasting were most prevalent. However, one of the prompts focused on foods eaten during the rainy season, which allowed perspectives across seasons to be included. Other potential limitations were sharing of cameras and the selection of photos for printing by those who shared a camera before the larger discussion. Although three participants shared a camera, they each took their own photos. Selection of photos for printing involved a discussion among the three women in the same age group, which helped to limit power dynamics in photo selection and also reduced duplicative photos. This process was also necessary because most of the participants in this study were not literate and needed assistance from the research assistant to write the captions for their photos. Finally, the photovoice prompts focused on food insecurity and did not specifically prompt participants to link food insecurity and child wasting. However, the participants were aware that this study was part of a project focused on wasting and connections between food insecurity and wasting emerged during discussions of the photos.

## 5 Conclusion

This study showed that across livelihoods, but particularly among pastoral and agropastoral groups, families struggled to procure enough food to adequately feed their children, especially during dry or lean seasons. Mothers perceived inadequate quantity and quality of food to be key contributors to child wasting, and they identified a number of barriers to achieving food security. To address these challenges, they recommended that shops should offer a better variety of foods, have longer opening hours, and maintain regular stock of food items. They wanted the county governments to improve infrastructure so they have better access to markets, provide inputs and training to enable kitchen gardening and ensure food availability, and work with donors to offer better quality relief food during droughts. The results of the photovoice sessions were discussed at county, sub‐county, and community levels together with findings from other components of the Nawiri longitudinal study to refine the implementation of Nawiri's multi‐sectoral program activities to address food insecurity and other factors that contribute to the persistent high prevalence of wasting in Turkana and Samburu Counties. Findings from this photovoice study also contribute to the growing photovoice literature on food insecurity and show the importance of including participatory approaches to identify community needs and potential solutions.

## Author Contributions

M.N.N. contributed to the interpretation of findings, supported data visualization, ensured compliance with ethical standards, drafted and revised the manuscript. V.L.F., E.M.S., F.T., C.W., C.L., A.W., and E.K.W. conceptualized and designed the study, supervised the research process, contributed to data analysis, contributed to the interpretation of findings, assisted in drafting and revising the manuscript. E.A., H.O., B.M., F.S., T.M. carried out data collection, conducted qualitative data analysis, contributed to the interpretation of findings, supported data visualization, ensured compliance with ethical standards, contributed to the final version of the manuscript.

## Conflicts of Interest

The authors declare no conflicts of interest.

## Supporting information


Supporting File

## Data Availability

The data are not publicly available due to privacy or ethical restrictions. The dataset is available from the African Population Health Research Center upon request.
