# Supplementary material for: Feeding Young Children in Food Insecure Environments: A Photovoice Study in Kenya's Drylands
Source: Matern Child Nutr. 2026 Oct 15;22(4):e70247. doi: 10.1111/mcn.70247 (PMC13670018; doi:10.1111/mcn.70247)
Supplement: Supplementary file 1 [file MCN-22-e70247-s001.docx]

**Nawiri Longitudinal Study:**

**Examining the Complex Dynamics Influencing Persistent Acute Malnutrition in Turkana and Samburu Counties – A Longitudinal Mixed Methods Study to Support Community Driven Activity Design**

**Photovoice Guide**

**DATE: __________________________**

**MODERATOR: __________________________**

**NOTE TAKER: ___________________________**

**START TIME: ________________	END TIME: __________________**

**INTERVIEW LANGUAGE:** **________________________**

**INTERVIEW CODE ________________________________________**

**Participant demographics:**

Age of Participant: ………………………………………………………………….

Sex of Participant: …………………………………………………………………..

Ethnic Group: …………………………………………………………………………

Education Level: ……………………………………………………………………..

Marital Status: ………………………………………………………………………..

Number of children under 0-5 years: …………………………………………

Religion: …………………………………………………………………………………

Occupation: …………………………………………………………………………….

Name of Village: ………………………………………………………………………

Livelihood zone: ………………………………………………………………………

**Discussion Guide**

Step 1: Match photos with numbers for data storage and easier identification during transcription.

Step 2: Remind participant on study objectives

**Topic List**

1. A place where you get or buy your food
2. The food you/family eats when the season has plenty or money is available
3. The types of food you/family consume when the season is lean, food prices are high or money is unavailable
4. Things that make it easy or difficult to attain your food basket

Step 3: Start discussion using participants’ pictures as the focus

I. Can you please pick the most important picture for you? ***Interviewer note****: Try and get a thorough description of the photos and make sure the participant has explained why the picture is important to understanding factors that influence child wasting.*

a) What do you see in this picture? For each photograph, tell me what it represents.
b) What is happening here? Please tell me about what the picture shows.
c) Why is this photograph important for understanding child wasting in your community?
d) How does this picture relate to your daily life?
e) Why does this practice or issue exist?
f) What makes it easy for families to do this? OR What makes it hard for families to do this?
g) If you had to write one sentence to tell the story of this photograph to a person looking at it in an exhibition what would it be?
II. Which picture do you want to talk about next? *(****Interviewer note****: Use this prompt for all other pictures left to discuss)*
III. Can you describe any other photos you would like to have taken to tell us about food insecurity and how it relates to child wasting in your village/community? What stopped you from taking them?
IV. If you could choose a photo to appear in an exhibit to tell a story about food insecurity in your community (facilitators or barriers), which one would it be? Why? *(****Interviewer note****: If the photo contains a face, check a photo release form exists, if not, choose another picture)*
V. Ask the participant to say (or write) a short caption/sentence that best describes what their picture is showing *(****Interviewer note****: Only ask this if the participant did not come up with a caption/one-sentence description when discussing this particular photo earlier).*
